# A Monoclonal Antibody-Based ELISA for Multiresidue Determination of Avermectins in Milk

**DOI:** 10.3390/molecules17067401

**Published:** 2012-06-15

**Authors:** Chunmei Wang, Zhanhui Wang, Wenxiao Jiang, Tiejun Mi, Jianzhong Shen

**Affiliations:** 1 Department of Pharmacology and Toxicology, College of Veterinary Medicine, China Agricultural University, Beijing 100193, China; 2 Key Laboratory of Detection for Veterinary Drug Residue and Illegal Additive, Ministry of Agriculture, Beijing 100193, China

**Keywords:** avermectins, monoclonal antibody, broad-selective determination, elisa, milk

## Abstract

Due to the widespread use and potential toxicity of avermectins (AVMs), multi-residue monitoring of AVMs in edible tissues, especially in milk, has become increasingly important. With the aim of developing a broad-selective immunoassay for AVMs, a broad-specific monoclonal antibody (Mab) was raised. Based on this Mab, a homologous indirect enzyme-linked immunosorbent assay (ELISA) for the rapid detection of AVMs in milk was developed. Under the optimized conditions, the IC_50_ values in assay buffer were estimated to be 3.05 ng/mL for abamectin, 13.10 ng/mL for ivermectin, 38.96 ng/mL for eprinomectin, 61.00 ng/mL for doramectin, 14.38 ng/mL for emamectin benzoate. Detection capability (CCβ) of the ELISA was less than 5 ng/mL and 2 ng/mL in milk samples prepared by simple dilution and solvent extraction, respectively. The optimized ELISA was used to quantify AVMs in milk samples spiked at different amounts. The mean recovery and coefficient of variation (CV) were 95.90% and 15.42%, respectively. The Mab-based ELISA achieved a great improvement in AVMs detection. Results proved this broad-selective ELISA would be useful for the multi-residue determination of AVMs in milk without purification process.

## 1. Introduction

Avermectins (AVMs) are insecticidal/miticidal compounds derived from the soil bacterium, *Streptomyces avermitilis.* Five AVMs (Table 1), namely abamectin (ABM), ivermectin (IVM), eprinomectin (EPR), doramectin (DOR) and emamectin (EMA) are widely used in agriculture and food-producing animals for the treatment of a broad spectrum of parasitic diseases. AVMs are very effective against parasites at extremely low doses. However, toxicology research has showed that an overdose of AVMs could cause a combination of clinical side effects ranging from mild to extremely severe, including death [1]. Given the potential hazard that AVMs pose to human and animal health, maximum residue limits (MRLs) are established in many countries. Joint FAO/WHO Expert Committee on Food Additives recommend the MRLs for IVM, DOR, and EPR in milk are 10, 15, 20 μg/L, respectively [2]. In the China, the MRL for IVM in milk is 10 μg/L, whereas the use of ABM and DOR is prohibited in cattle producing milk for human consumption [3].

**Table 1:** Chemical structures of avermectins ^a^. 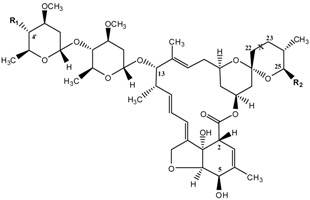

Substance	Abbreviation	X(C_22_ – C_23_)	R_1_	R_2_
Abamectin	ABM	–CH=CH–	OH	CH(CH_3_)Y ^b^
Ivermectin	IVM	–CH_2_–CH_2_–	OH	CH(CH_3_)Y ^b^
Eprinomectin	EPR	–CH=CH–	NHC(O)CH_3_	CH(CH_3_)Y ^b^
Doramectin	DOR	–CH=CH–	OH	C_6_H_5_
Emamectin	EMA	–CH=CH–	NH–CH_3_	CH(CH_3_)Y ^b^

^a^ Each drug has two homologues, with the major component comprising more than 80% (B1a) and the minor less than 20% (B1b) of the AVMs. The major component is used as the marker compound to calculate total residues in edible tissues; ^b^ Component B1a–Y=C_2_H_5_, component B1b–Y=CH_3_.

The conventional methods for detection of AVMs involving high performance liquid chromatography (HPLC) and liquid chromatography/mass spectrometry (LC/MS) are sensitive and reliable [4,5,6,7]. However, these applications are relatively time-consuming and usable only on a laboratory scale with expensive instruments. Since immunoassays have proven to be a powerful tool for high throughput and on-site screening analysis for surveillance and monitoring purpose in a wide variety of food matrices [8], broad-selective immunodetection prior to chromatographic determination may be an attractive approach for multi-residue monitoring. If the total quantity of two or more AVMs detected in a sample is less than the MRL, the sample needs no further inspection.

Immunochemical methods involve the use of antibodies, which are the key components of all immunoassays because their quality greatly contributes to the sensitivity and selectivity [9]. Some antibodies have been produced against single AVM [10,11,12,13,14,15,16], whereas few broad-specific antibodies have been reported. Schmidt *et al.* [17] described a monoclonal antibody (Mab) with high specificity, but the Mab could recognize only two AVMs. In our previous work [18], a broad-selective ELISA for three AVMs using a polyclonal antibody (Pab) was developed. Since Mab offered a more definite specificity than Pab and an unlimited production, in this work, a broad specific Mab was produced. Moreover, a broad-selective ELISA based on this Mab was developed, and applied to detect all the five AVMs in milk samples.

## 2. Results and Discussion

### 2.1. Antigen Synthesis

AVMs are small molecules and must be conjugated to a carrier protein to elicit an immune response. The design of the required immunogen is critical for the success of rapid immunoassay. AVMs have three available hydroxy groups (Table 1), and especially the 4′-OH can be modified to conjugate with a carrier protein for the production of antibodies against AVMs [17]. Moreover, 4′-*O*-succinoyl-ABM had been successfully used to produce broad-specific Pab against AVMs in our previous research [18]. In this study, the same immunogen was used for the Mab production, and the structure of ABM was used as the determinant group.

For assay purposes, 4′-*O*-succinoyl-ABM-OVA, 5-ABM-OVA and 5-ABM oxime-OVA were synthesized as coating antigens. Conjugate formation was confirmed spectrophotometrically. UV-Vis spectra showed qualitative differences between the carrier protein and conjugates in the region of maximum absorbance of ABM. Molar ratios of the conjugates ranged from 2 to 15.

### 2.2. Mab Production and Characterization

Since a single B-lymphocyte produces a single type of antibody molecule, screening for the hybridomas which could secrete broad specific Mabs, would be a pivotal process. In order to choose broad specific Mabs, indirect ELISA for IVM detection was performed to screen hybridomas. Five cell fusion experiments were conducted. After screening, two selected Mabs (named 2C11, 3A9) showed good recognition towards IVM. The hybridomas were injected intraperitoneally into mineral oil-primed mice to produce ascites and then purified. Their sensitivity and specificity were investigated by indirect ELISAs using three coating antigens (Table 2). Neligible CR was observed from moxidectin, which belongs to milbemycins family and the structure is similar to AVMs but lacks the disaccharide group. Sensitivity results of ABM with the two Mabs and three coating antigens were very similar (IC_50_ values ranging from 38.97 to 65.62 ng/mL). When the CR of ABM was set as 100%, broad selectivity was evaluated by the other four AVMs. The combination of Mab 2C11 and 4′-*O*-succinoyl-ABM-OVA was selected as the optimal assay for broad selective detection, with which the CR values were 50.46%, 14.17%, 19.82% and 47.81% for IVM, EPR, DOR and EMA benzoate, respectively.

**Table 2:** Characterization of the selected Mabs ^a^.

Mab	Coating antigen	IC_50_ (ng/mL)	Cross reactivity (%)			
		ABM	IVM	EPR	DOR	EMA benzoate
2C11	4′- *O*-succinoyl-ABM-OVA	45.13	**50.46**	**14.17**	**19.82**	**47.81**
2C11	5-ABM-OVA	38.97	37.69	10.33	14.55	30.66
2C11	5-ABM oxime-OVA	65.54	47.07	16.98	19.12	50.11
3A9	4′- *O*-succinoyl-ABM-OVA	50.36	26.84	7.89	21.48	25.43
3A9	5-ABM-OVA	54.31	61.20	7.11	10.86	32.75
3A9	5-ABM oxime-OVA	65.62	16.85	10.93	13.87	19.17

^a^ PBS was used to dissolve the Mab and standards, the incubation temperature was 37 °C.

### 2.3. Assay Optimization

Immunoassays are usually carried out under physiological conditions, and frequently influenced by several nonspecific parameters such as temperature, time, pH, ionic strength, and presence of organic solvent. With the aim of improving immunoassay performance, the influence of several nonspecific parameters on assay characteristics was examined. Maximal absorbance (A_max_)/IC_50_ ratio was a convenient estimate of the influence of some factors on the ELISA sensitivity, the higher ratio indicating higher sensitivity [21].

#### 2.3.1. Assay Buffer

Four combinations of Mab dilution buffer and standard preparation buffer were performed to investigate the effect of assay buffer. As shown in Table 3, the IC_50 _value was significant increased when ABM standards were dissolved in PBS, which implied that the concentration of K^+^ had a great impact on the ELISA property. Apart from A_max_/IC_50_ ratio, dynamic range, and limit of detection were also taken into account. Therefore, PB was selected to dissolve both Mab and standards.

**Table 3:** A_max_ and IC_50_ values of ABM standard curves in different incubation buffers.

Mab dilution buffer	Standard preparation buffer	A_max_^a ^(OD_450nm_)	IC_50 _(ng/mL)
PBS	PBS	1.98	42.34
PB	PBS	2.10	27.94
PBS	PB	1.81	8.31
PB	PB	1.78	4.82

^a^ Results were the means of four determinations performed in the same ELISA plate.

#### 2.3.2. pH Effect

**Figure 1:**
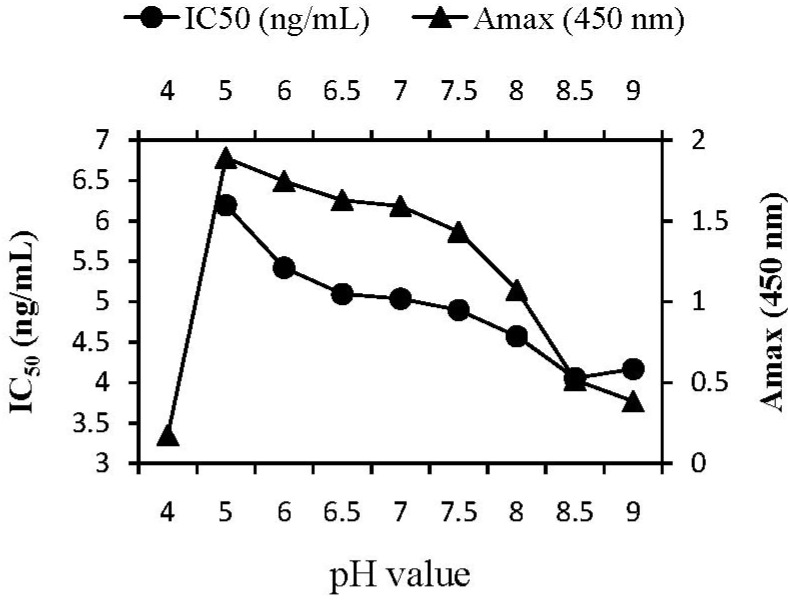
Effect of pH on ELISA performance. For the competition step, standards and Mab were dissolved in PB of different pH, and the GαM^HRP^ was diluted in PB of pH 7.1. Incubation temperature was 37 °C.

The plots of assay parameters (A_max_, IC_50_) as a function of pH value were depicted in Figure 1. The ability of Mab to recognize the coating antigen (A_max_) decreased gradually (from 1.89 to 0.38) as pH value increased from 5 to 9. In addition, the recognition of ABM (IC_50_) did not change markedly (from 6.19 to 4.06 ng/mL) until pH value increased to pH 9. On the basis of the result that maximal A_max_/IC_50_ ratio was 0.32 for pH 6, followed pH 6.5 and of the fact that antigen-antibody reaction was more stable in neutral environments, pH 6.5 seemed to be a reasonable choice for the competition step.

#### 2.3.3. Methanol Concentration

**Figure 2:**
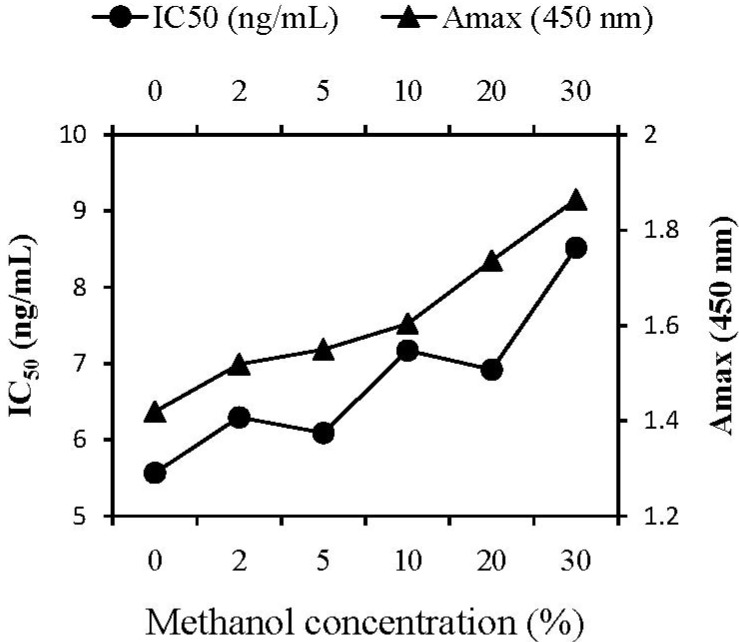
Effect of methanol concentration on ELISA performance. For the competition step, standards were prepared in PB (pH 6.5) containing different concentration of methanol and the Mab was diluted in PB (pH 6.5), incubation temperature was 37 °C.

Since AVMs are highly lipophilic, and organic solvents are often used to extract analytes from samples in the immunoassays, it is necessary to use a water-miscible organic solvent in the assay buffer for ELISAs. Methanol was the most popular solvent for immunoassays [22,23], and 10% methanol was selected previously for the Pab-based ELISA against AVMs [18]. In this work, the effect of solvent on the ELISA performance was evaluated by preparing standard curves in buffers containing various concentration of methanol (Figure 2). On the one hand, maximum absorbance went up as methanol concentration increased. On the other hand, the immunoassay showed a tendency to decrease the sensitivity of the immunoassay (lower IC_50_ value) with increase of methanol concentration. Judged by the A_max_/IC_50_ ratio, the optimal concentration of methanol in buffer was 5%.

#### 2.3.4. Incubation Temperature

**Figure 3:**
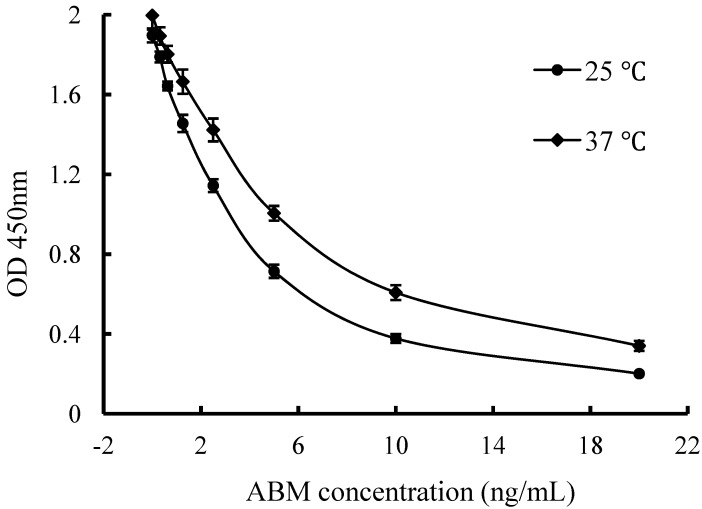
Effect of incubation temperature on ELISA performance. For the competition step, standards were prepared in 5% methanol-PB (pH 6.5) and the Mab was diluted in PB (pH 6.5). Each point represents the average of five replicates.

Both temperature and time are important physical parameter for the immunoassay. In general terms, the higher temperature was, the shorter the incubation time needed, and an increase of the incubation time resulted in higher maximum absorbance [24,25]. To investigate the effect of incubation temperature on ELISA performance, the incubation time was set at 30 min, and competitive curves were performed at both 37 °C and 25 °C. As shown in Figure 3, higher sensitivity was observed when the incubation temperature was at 25 °C. Moreover, in the case of incubation at 37 °C, the intra-assay coefficient of variation (CV) increased evidently. Therefore, incubation temperature of 25 °C was selected for further study.

Taking all these factors into account, the optimized conditions for the AVMs immunoassay summarized as follows: The coating antigen was 4′-*O*-succinoyl-ABM-OVA (diluted to 1:10,000), Mab was 2C11 (diluted to 1:70,000), room temperature (25 °C) for all incubations, overnight incubation (4 °C) for the coating step, an incubation time of 30 min for the competitive step, PB buffer (10 mM, pH 6.5) for Mab dilution and 5% methanol-PB for standard preparation.

**Figure 4:**
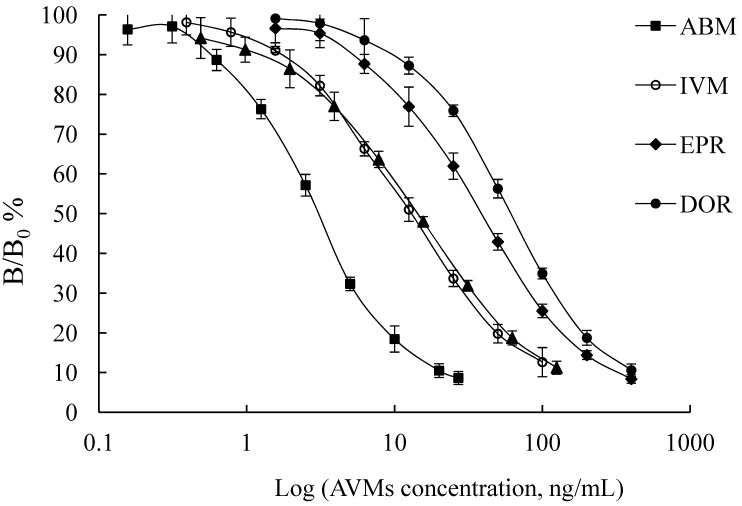
Standard curves of the five AVMs in assay buffer. Each point represents the average of five determinations performed in different ELISA plates.

ELISAs were performed under the above-selected conditions to establish standard curves for five AVMs. As seen from Figure 4, the IC_50_ value in assay buffer was 3.05 ng/mL for ABM, 13.10 ng/mL for IVM, 38.96 ng/mL for EPR, 61.00 ng/mL for DOR and 14.38 ng/mL for EMA benzoate. Concerning the reproducibility, average intra-assay CVs of the IC_50_ value were 4.87%, 12.01%, 7.93%, 6.48% and 9.12% for ABM, IVM, EPR, DOR and EMA benzoate, respectively. The assay for ABM was the most sensitive compared with the literature [16,17,18], and the sensitivity for IVM was also higher than those reported in Refs. [12,13,14,18,20]. In our previous research [18], it was observed that EPR could be well recognized by the Pab, in which CR was 145.40% (CR for ABM was set as 100%), whereas in this work, the weak recognition of EPR (CR = 7.82%) could be attributed to a more definite specificity of the Mab than the Pab. On the other hand, EMA benzoate could be also recognized by the Mab (CR = 21.21%), which meant that the Mab had broader selectivity than the Pab.

### 2.4. Milk Analysis

#### 2.4.1. Sample Preparation by Simple Dilution

**Figure 5:**
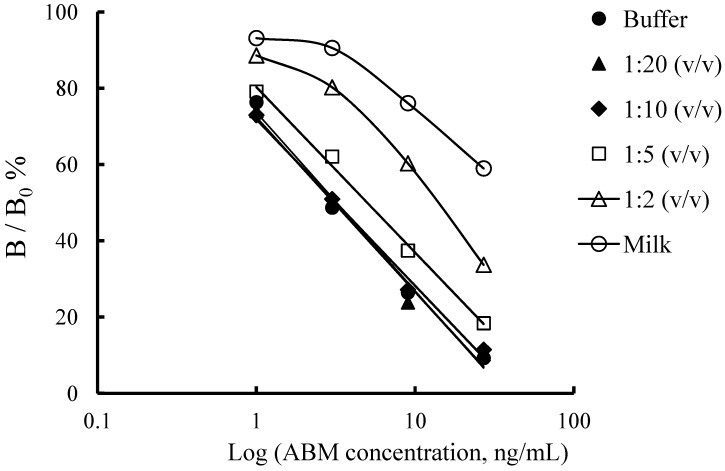
ELISA standard curves for ABM in matrix of milk sample. Milk samples were diluted by 5% methanol-PB (pH 6.5) and incubation temperature was 25 °C.

Analysis of real samples by immunoassay usually requires some sort of sample pretreatment to circumvent the matrix effect. Dilution, which was considered as an effective means [26], significantly decreased matrix interference of samples, though it simultaneously caused reduction of assay sensitivity due to the shift of the dynamic range. In order to investigate the effect of milk matrix on the ELISA performance, different dilution factors were used to prepare ABM standard curves, and total factors were set comparing with the standard curve in buffer. As shown in Figure 5, the assay parameters showed no noticeable difference between ABM standard curves obtained at a dilution of 1:10 (v/v) and in buffer. Therefore, to balance the sensitivity and the dynamic range of the immunoassay, milk samples were diluted to 1:10 (v/v) for detection of AVMs.

To evaluate the detection capability of the ELISA, twenty blank samples were selected and replicates of the samples were spiked with ABM at 5 ng/mL, and the blank samples and the spiked samples were determined simultaneously by the developed ELISA. Results showed that none of the responses of the spiked samples overlap with the range of responses of the blank samples. Thus the detection capability (CCβ) of the ELISA was less than 5 ng/mL in milk with simple dilution preparation.

The accuracy and precision of the ELISA were represented by recovery and coefficient of variation (CV), respectively (Table 4). To evaluate the LOD of the ELISA, twenty blank samples were systematically included in the analysis. For IVM and EPR, the values of blank samples were obviously lower than the lowest concentration of the corresponding standard curve. Thus the LOD for IVM and EPR was 7.81 and 15.60 ng/mL, respectively. On the other hand, based on the mean value of twenty blank samples plus three times of the mean standard deviation, the LOD for ABM, DOR and EMA benzoate was 3.97, 30.05 and 11.93 ng/mL, respectively. The mean recoveries at two spiked levels were 83.29%, 101.31%, 88.56%, 75.65% and 101.73% for ABM, IVM, EPR, DOR and EMA benzoate, respectively. The mean intra-assay CVs for ABM, IVM, EPR, DOR and EMA benzoate were 19.28%, 13.39%, 10.17%, 15.71% and 12.97%, respectively. Moreover, the mean inter-assay CV was also satisfactory, which was 14.10% in average and ranged from 6.40% to 20.64%. These results confirmed that the established Mab-based ELISA could detect IVM and EPR at their MRL levels (CAC, 2011) in milk sample with simple dilution preparation.

**Table 4:** Recoveries of AVMs from spiked milk samples with simple dilution preparation (n = 4).

Analyte	Spiked (ng/mL)	Intra-assay		Inter-assay	
		Recovery (%)	CV (%)	Recovery (%)	CV (%)
ABM	5	83.13 ± 23.07	28.32	89.65 ± 13.45	15.31
	10	86.02 ± 8.62	10.23	74.34 ± 4.66	6.40
IVM	10	121.12 ± 16.90	14.24	113.75 ± 23.00	20.64
	20	101.32 ± 12.45	12.54	69.03 ± 13.26	19.61
EPR	20	88.23 ± 11.94	13.81	88.16 ± 13.31	15.41
	40	73.02 ± 4.67	6.53	104.81 ± 11.27	10.97
DOR	50	94.15 ± 20.55	22.28	75.53 ± 13.16	17.78
	100	63.73 ± 5.70	9.13	69.19 ± 11.29	16.65
EMA benzoate	15	100.50 ± 16.38	16.63	102.62 ± 9.83	9.77
	30	104.46 ± 9.52	9.30	99.33 ± 8.27	8.50

#### 2.4.2. Sample Preparation by Solvent Extraction

To improve the detection capability of the ELISA in milk, the dilution factor was decreased by solvent extraction in sample preparation. The assay parameters showed no noticeable difference between ABM standard curves obtained in assay buffer and in extracted milk matrix (dilution factor was 1:4, v/v). Based on the mean value of twenty blank samples plus three times of the mean standard deviation, the LODs for ABM, IVM and EMA benzoate were 1.28, 2.94 and 3.15 ng/mL, respectively. For EPR and DOR, the values of blank samples were obviously lower than the lowest concentration of the corresponding standard curve. Thus the LOD for EPR and DOR was 6.24 and 12.50 ng/mL, respectively. When twenty blank samples were selected and replicates of the samples were spiked with ABM at 2 ng/mL, none of the responses of the spiked samples overlapped with the range of responses of the blank samples. Therefore, when milk was prepared by solvent extraction, the CCβ of the ELISA was less than 2 ng/mL. Additionally, milk samples were also analyzed by the ELISA after being fortified with AVMs. As shown in Table 5, the mean recoveries at two spiked levels were between 69.05% and 130.89%. The mean assay CV, which was 16.63% on average and ranged from 7.08% to 28.40%, was also satisfactory. These results confirmed that the established Mab-based ELISA was a potential screening tool for AVM monitoring in milk samples.

**Table 5:** Recoveries of AVMs from spiked milk samples with solvent extraction (n = 4).

Analyte	Spiked (ng/mL)	Intra-assay		Inter-assay	
		Recovery (%)	CV (%)	Recovery (%)	CV (%)
ABM	2	106.60 ± 13.00	12.44	94.92 ± 20.27	21.79
	4	124.18 ± 8.93	7.33	112.39 ± 31.28	28.40
IVM	5	114.84 ± 22.83	20.29	116.95 ± 15.93	13.90
	10	75.00 ± 16.57	22.55	95.72 ± 19.59	20.89
EPR	10	87.50 ± 13.26	15.46	69.05 ± 8.22	12.15
	20	117.49 ± 14.55	12.64	84.19 ± 12.16	14.74
DOR	15	130.89 ± 13.56	10.57	85.58 ± 21.56	25.70
	30	106.60 ± 7.39	7.08	106.88 ± 13.92	13.29
EMA benzoate	5	98.14 ± 14.50	15.07	100.71 ± 18.98	19.23
	10	92.01 ± 24.91	27.63	114.28 ± 12.91	11.52

## 3. Experimental

### 3.1. Reagents

ABM (purity 98.27%) and DOR (purity 94.33%) were obtained from Pfizer Co. (New York, NY, USA), IVM (purity 95.64%) and EMA benzoate (purity 98.26%) were sourced from CAU Newtech Development Co. (Beijing, China), and EPR (purity 96.85%) was a gift from Prof. Ming Wang (China Agricultural University, Beijing, China).

Ovalbumin (OVA, MW45000), bovine serum albumin (BSA, MW67000), peroxidase-labeled goat anti-mouse immunoglobulins (GαM^HRP^), culture media RPMI-1640, hypoxanthine-aminopterin-thymidine (HAT) and hypoxanthine-thymidine (HT) medium supplements, pristane, complete and incomplete Freund’s adjuvants, dimethyl sulfoxide (DMSO) and polyethylene glycol 3350 were purchased from Sigma-Aldrich (Madrid, Spain). SP2/0 mouse plasmacytoma line was from the Shanghai Institute of Cell Biology (Shanghai, China). 3,3′,5,5′-Tetramethylbenzidine (TMB), and Tween-20 were purchased from Shanghai Chemical Reagents Company (Shanghai, China). All of the other chemicals and organic solvents were of analytical grade.

### 3.2. Instrumentation

Ultraviolet-visible (UV-vis) spectra were recorded on a spectrophotometer (Xinmao, Shanghai, China). 96-well and 24-well cell culture plates, 96-well polystyrene microplates were purchased from Costar (Costar Inc., Milpitas, CA, USA). Immunoassay absorbance was read with a Multiscan Spectrum purchased from Thermo (Labsystems, Vantaa, Finland) in dual wavelength mode (450–650 nm).

### 3.3. Buffers

Unless otherwise indicated, PBS was10 mM phosphate-buffered saline (containing 137 mM NaCl and 2.7 mM KCl, pH 7.4). Coating buffer was 50 mM carbonate–bicarbonate buffer (CB, pH 9.6). PBST was PBS with 0.05% Tween 20. Blocking buffer was PBS with 0.5% casein and 5% fetal calf serum (FCS). PB was 10 mM phosphate buffer (containing 274 mM NaCl and 5.4 mM KCl, pH 7.1). Citrate buffer was a 40 mM solution of sodium citrate (pH 5.5). The substrate solution contained 0.01% TMB and 0.004% H_2_O_2_ in citrate buffer.

### 3.4. Immunogen and Coating Antigens

4′-*O*-succinoyl-ABM-BSA (immunogen) and 4′-*O*-succinoyl-ABM-OVA had been prepared in a previous study [18]. According to Mitsui *et al.* [10], 5-*O*-[(4-nitrophenyl)oxyl carbonyl]-ABM and ABM oxime were conjugated to OVA to prepare 5-ABM-OVA and 5-ABM oxime-OVA as coating antigens.

### 3.5. Mab Production and Characterization

BALB/c female mice (8–10 week old) were immunized with 4′-*O*-succinoyl-ABM-BSA conjugate. Immunizing strategy and cell fusion procedure were the same as that we reported before [19]. Twelve to fourteen days after cell fusion, culture supernatants were screened with the coating antigen 4′-*O*-succinoyl-ABM-OVA for the presence of antibodies that could recognize IVM. Selected hybridomas were subcloned by limiting dilution. Stable antibody-producing clones were expanded and stored in liquid nitrogen. Hybridomas that could produce Mabs with broad-selectivity and high sensitivity were injected intraperitoneally into pristane-primed mice to produce ascites. Then, Mabs were separated and purified by salt precipitation (with caprylic acid-ammonium sulfate) as described by Svendsen *et al.* [20]. Selectivity of these Mabs was investigated by indirect competitive ELISA using all the three coating antigens. And the cross-reactivity (CR) was calculated by the following equation:


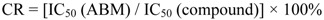


### 3.6. Competitive Indirect ELISA

For competition assays, the concentrations of antibody and coating antigen were optimized by checkerboard titration. After each step, plates were washed four times with PBST. The ELISA was run as described in a previous paper [18]. Briefly, microtiter plates were coated with the optimized concentrations of antigens in CB (100 µL/well) by incubation at 4 °C overnight. Nonspecific binding sites were blocked with the blocking buffer (200 µL/well) at 37 °C for 2 h. Afterward, serial dilutions (50 µL/well) of the analyte were added, followed by adding 50 µL/well of Mab at a previously determined concentration. The mixture solution was allowed to incubate at 37 °C for 30 min and then 100 µL per well of diluted (1/10,000) GαM^HRP^ was added. After another 30 min incubation, 100 µL/well of substrate solution was added. After incubation for 15 min, the reaction was stopped by adding 50 µL of 2 M H_2_SO_4_ and absorbance at 450 nm was measured.

Competitive curves were obtained by plotting inhibition (inhibition = B/B_0_ × 100%) against the logarithm of analyte concentration. Sigmoid curves were simulated by means of Origin 7.0 software. From the equations, IC_50_ values (*i.e*., analyte concentrations at which the binding of the antibody to the coating conjugate were inhibited by 50%) were determined to assess the assay sensitivity. 

### 3.7. ELISA Optimization

*Assay buffer.* The effect of buffering capacity of assay solution on ELISA performance was studied using PB and PBS to dissolve Mab and ABM standards. Four competitive curves were performed with above Mab and ABM standards to assess the effect of the assay buffer (PB and PBS).

*pH effect*. PBs were adjusted to different pH values (4.0–9.0) that were prepared by changing the amounts of Na_2_HPO_4_ and NaH_2_PO_4_, whereas the concentrations of NaCl and KCl remained at 274 and 54 mM, respectively. These buffers were used to prepare ABM standard solutions and the antibody solutions that were employed for the competitive ELISA to obtain the standard curves.

*Methanol concentration*. The assays’ tolerance to organic solvent was evaluated between 0 and 30% methanol concentration (v/v). In this case, competitive curves were performed from the ABM standards dissolved in PB (pH 6.5) containing different amounts of methanol and Mab in PB (pH 6.5).

*Incubation temperature*. Competitive curves were performed from the ABM standards dissolved in 5% methanol-PB (pH 6.5) and Mab in PB (pH 6.5). The incubation temperature was set at 37 °C and 25 °C to assess the influence of incubation temperature.

### 3.8. Milk Analysis

Different milk samples were purchased from local markets. All the control samples were previously checked as AVMs free using HPLC method at the National Reference Laboratory for Veterinary Drug Residue (Beijing, China).

Two sample pretreatment procedures were performed. Simple dilution: To investigate the effect of milk matrix on assay performance, milk sample (0.5 mL) was diluted to different volumes (0.5, 1, 2.5, 5 and 10 mL) with 5% methanol-PB (pH 6.5). Then 50 μL portions were used for the selected ELISA test to investigate the matrix interference on the ABM standard curve. For spiked samples, 1 mL of standard solution was spiked to 10 mL milk sample, and diluted to 1:10 (v/v) by 5% methanol-PB (pH 6.5). Solvent extraction: Milk sample (3 mL) was added acetonitrile (9 mL) and hexane (3 mL), and the mixture was shaken for 1 min with a vortex mixer. After centrifugation (4,000 rpm, 10 min), a portion of the acetonitrile layer (1 mL) was transferred and evaporated at 50 °C under a nitrogen stream. The dry residue was dissolved in 5% methanol-PB (pH 6.5, 1 mL). Determination of spiked samples was performed by interpolating their mean absorbance of triplicates in the standard curve run on the same plate.

The assay validation of the ELISA was carried out according to the related content of Commission Decision 2002/657/EC and guidelines for the validation of screening methods for residues of veterinary medicines. The detection capability (CCβ) is the smallest content of the analyte that may be detected, identified and/or quantified in a sample with an error probability of β (<5%).

The limit of detection (LOD) was defined as the lowest amount of analyte in a sample that could be detected but not necessarily quantified exactly, which was based on the mean value of 20 blank samples plus three times of the mean standard deviation. The accuracy and precision of the method were represented by recovery and coefficient of variation (CV), respectively. The precision of the ELISA method was analyzed by repeated analysis of the spiked samples and comparison of the intra- and inter-assay CVs. Intra-assay variation was measured by four replicates of each spiked concentrations. And the inter-assay variation was based on the results of four different days.

## 4. Conclusions

A broad-specific Mab (2C11) for AVMs was produced. The optimized Mab-based indirect competitive ELISA was sensitive and accurate. Recovery results proved this broad-selective ELISA would be useful for the multi-residue determination of AVMs in milk.
